# Maximum resection and immunotherapy improve glioblastoma patient survival: a retrospective single-institution prognostic analysis

**DOI:** 10.1186/s12883-021-02318-1

**Published:** 2021-07-19

**Authors:** Eiichi Ishikawa, Narushi Sugii, Masahide Matsuda, Hidehiro Kohzuki, Takao Tsurubuchi, Hiroyoshi Akutsu, Shingo Takano, Masashi Mizumoto, Hideyuki Sakurai, Akira Matsumura

**Affiliations:** 1 Department of Neurosurgery, Faculty of Medicine, University of Tsukuba, 1-1-1 Tennodai, Tsukuba, Ibaraki 305-8575 Japan; 2 Department of Radiation Oncology, Proton Medical Research Center, University of Tsukuba, Tsukuba, Ibaraki Japan

**Keywords:** Tumor removal, Glioma, Intraoperative MRI, Immunotherapy

## Abstract

Glioblastoma (GBM) is a refractory disease with a poor prognosis and various methods, including maximum resection and immunotherapy, have been tested to improve outcomes. In this retrospective study we analyzed the prognostic factors of 277 newly diagnosed GBM patients over 11 years of consecutive cases at our institution to evaluate the effect of these methods on prognosis. Various data, including the extent of removal (EOR) and type of adjuvant therapy, were examined and prognostic relationships were analyzed. The median overall survival (OS) of the entire 277-case cohort, 200 non-biopsy cases, and 77 biopsy cases was 16.6 months, 19.7 months, and 9.7 months, respectively. Gross total removal (GTR; 100% of EOR) was achieved in 32.9% of the cases. Univariate analysis revealed younger age, right side, higher Karnofsky performance status, GTR, intraoperative magnetic resonance imaging (MRI) use for removal, proton therapy, combination immunotherapy, and discharge to home as good prognostic factors. Intraoperative MRI use and EOR were closely related. In the multivariate analysis, GTR, proton therapy, and a combination of immunotherapies, including autologous formalin-fixed tumor vaccine, were the significant prognostic factors. A multivariate analysis of 91 GTR cases showed that immunotherapy contributed to prognostic improvements. The median OS and 5-year OS % values were 36.9 months and 43.3% in GTR cases receiving immunotherapy. In conclusion, GTR, proton therapy, and immunotherapy were good prognostic factors in single-center GBM cases. Tumor vaccine therapy for GTR cases achieved a notably high median survival time and long-term survival ratio, indicating its usefulness in GTR cases.

**Supplementary Information:**

The online version contains supplementary material available at 10.1186/s12883-021-02318-1.

## Introduction

Glioblastoma (GBM) is generally a refractory disease with a poor prognosis for the majority of patients [1]. Past papers have shown prognosis-related factors in GBM patients receiving standard radiochemotherapy, such as the extent of removal [2–4], patient age, isocitrate dehydrogenase (IDH) mutations, and methylation status of O6-methylguanine-DNA methyl-transferase (MGMT) promotor among others [5–7]. In addition, multiple immunotherapies for GBM patients have been conducted in an attempt to improve prognoses [8, 9]. Since January 2013, we have utilized intraoperative magnetic resonance imaging (MRI) to improve the extent of removal [10, 11] in addition to advanced treatments, including immunotherapy and proton therapy [12–15]. In March 2013, we started a double-blinded, randomized phase IIb/III trial of autologous formalin-fixed tumor vaccine (AFTV) with standard chemotherapy and temozolomide (TMZ) for newly diagnosed GBM to evaluate the efficacy of AFTV for prevention of recurrence and/or cure of residual tumor burden (UMIN000010602) [13] and, since October 2020, GBM patients who had gross-total removal (GTR), resulting in no residual gadolinium enhancement, or subtotal removal (STR) are enrolled in a double-blinded, randomized phase III trial of AFTV. Similarly, in a double-blinded, randomized phase II trial of dendritic cell vaccine ICT-107, GBM patients who had undergone GTR, resulting in no residual gadolinium enhancement, or STR were enrolled. [16] In that study, progression-free survival (PFS) in the intent-to-treat (ITT) population was significantly increased in the ICT-107 cohort by 2.2 months. In a phase 3 trial of autologous tumors featuring lysate-pulsed, dendritic cell vaccine (DCVax®-L) with standard therapy for newly diagnosed GBM, 63% of enrolled cases were GTR cases [17] and the median overall survival (OS) of the overall ITT population (according to blinded interim data) was 23.1 months from the time of surgery. Moreover, various clinical methodologies, such as combining immune checkpoint inhibitors to overcome relapse-mediating immunosuppressive mechanisms in the GBM microenvironment after monotherapy, are being tested [8]. However, none of the currently published papers examines the superiority of immunotherapies in a phase III clinical study.

Thus, the purpose of this study is to analyze the prognostic factors in consecutive cases of newly diagnosed GBM in our institution for the past 11 years during the TMZ era and evaluate both the extent of removal and the effect of these advanced treatments on GBM cases.

## Methods

### Patient enrollment

A total of 277 patients with newly diagnosed GBM who were treated in our institution from June 2009 to March 2020, including previous clinical trials at our institution, were registered in this retrospective study. Although TMZ was approved in Japan in September 2006, nimustine (ACNU) was widely used until May 2008 at our institution, while many patients had unknown prognoses up through May 2009. Therefore, all patients until May 2009 at the same time with both ACNU-receiving and patients with unknown prognosis were excluded. The median follow-up periods for all patients and living patients were 14.7 (0.5–118.2, the minimal and the maximal values) and 16.0 (2.3–82.2) months, respectively. In this study, a small number of IDH-mutant GBM cases were included but secondary GBM patients who had previously received any treatment and/or follow-up as lower-grade glioma patients or primary GBM patients who were previously treated in any institution before surgery were excluded. The study protocol was approved by the Ethics Committee of the University of Tsukuba Hospital, Japan (number R01-202). Opt-out consent published on the website of our institution was used for enrolled patients to protect individual privacy while the requirement to obtain written informed consent was waived by this Ethics Committee. The study was carried out in accordance with relevant guidelines and regulations.

Various data, including patient age, sex, the main location of the tumor, tumor side, Karnofsky Performance Status (KPS) of patients, the extent of removal (EOR), intraoperative photodynamic diagnosis (PDD) using 5-aminolevulinic acid (5-ALA), use of intraoperative MRI, IDH status, p53 status, MGMT status, type of radiotherapy, type of adjuvant therapy, and patient return at discharge were examined for relationships to the prognoses. As a result, MGMT promotor statuses data were excluded from this univariate analysis because the measurement method differed from time to time and there were many missing values. As for EOR evaluated with T1weighted-images after gadolinium administration on MRI within 3 days after surgery, GTR for the complete disappearance of the contrast area (100% of EOR), STR for tumor volume reduction of 90% or more, or tumor residuals with a maximum diameter of 10 mm or less, partial removal (PR) for 5% or more and less than 90%, and biopsy for less than 5% were set as fixed definitions. In our institution, a biopsy or PR was selected for some patients with GBM located in an unresectable area or high-risk region (e.g., in the case of deep-seated lesions) as shown in Supplementary Table 1. Additionally, older patients often revoked consent for maximal resection, which resulted in biopsy or PR. Information on the PDD fluorescence intensity was collected from surgical records and classified as strong, vague, or negative, and those cases with positive fluorescence intensity but without any detailed descriptions were classified as positive.

The timing of recurrence in this study was determined based on the medical record entries. At our institution, patients receive outpatient consultations every month and undergo MRI every 2–3 months or when symptoms appear. The tumor growth on MRI is basically evaluated using the Response Assessment in Neuro-Oncology criteria. In case of suspected recurrence, treatment is continued, and thorough follow-up by MRI and/or 11C-methionine positron emission tomography (PET) is performed to confirm the recurrence.

### Intraoperative MRI system for tumor removal or biopsy and postoperative treatments

Our intraoperative MRI system, the VISIUS® Surgical Theatre (IMRIS, Minnetonka, MN, USA) features both high-field (1.5 T) ceiling-mounted and movable magnets [10] to confirm the presence or absence of residual tumors for cases with maximum removal or to confirm accurate positioning for biopsy. Our institution uses the ceiling-mounted movable magnet both for removal and biopsy. For postoperative radiotherapy (RT), extended local radiation using 60 Gy (30 fractions) total irradiation dose or similar protocols were classified as ‘conventional RT’, RT using 45 Gy (15 fractions) or similar protocols were ‘hypo-fractionated RT’, and RT using proton beams or combined with proton beams were classified as ‘proton therapy’. The conventional RT was typically administered 3 to 5 weeks after tumor removal in GBM patients, and proton therapy was selected when indicated and desired by the patient. The critical inclusion criteria for proton therapy were as follows: predicted radiation necrosis was unlikely to be fatal, the potential resectability of a lesion when brain necrosis was found in the 96.6 Gy irradiation range [15], and patients who accepted the advanced therapy after studying the informed consent that fully and carefully explained possible complications. In principle, hypo-fractionated RT was selected for elderly patients with low KPS. In addition, rare cases of whole-brain radiotherapy (WBRT) were grouped separately.

For postoperative adjuvant therapy, cases treated according to the modified Stupp regimen (RT concomitant with TMZ followed by TMZ maintenance therapy until recurrence or for 12 to 24 times) were classified into the ‘TMZ’ group. Cases using immunotherapy in addition to the modified Stupp regimen were classified into the ‘immunotherapy’ group. The implementation of these immunotherapies and the types of immunotherapies largely depends on the timing of the clinical research. Of the 39 cases in the immunotherapy group at our institution, 31 cases received autologous formalin-fixed tumor vaccine (AFTV), the manufacturing method of which is described in our previous paper [12], 2 cases received interferon beta, and 6 cases received other drugs. Three of these cases with AFTV also received proton therapy. The critical inclusion criteria for the AFTV therapy were as follows: patients who underwent maximum possible resection (at least non-biopsy surgery) of the tumor, at least 1.5 g of neoplastic tissue for AFTV preparation was available [12], and patients who accepted the advanced therapy after careful reading of the informed consent that fully explained possible complications. Patients who started bevacizumab (BEV) before or concomitant with RT were classified into the ‘bevacizumab’ group. Our institution’s clinical protocol indicates that BEV treatment be used for low KPS patients receiving biopsy or PR surgery, usually with hypo-fractionated RT, and most BEV cases also used TMZ.

### Detection of p53 and IDH statuses

For immunohistochemistry (IHC) surveys of p53 statuses, the corresponding staining indices were calculated as the average number of positive cells in the best-stained tumor areas with a total number of cells not less than 1000, as described previously [12]. For category analyses, cases with 10% or more positive cells were rated as positive, while cases with fewer than 10% positive cells were rated as negative for p53 [12]. For p53 status, positive status, as determined by IHC in our institute, and mutation results from Sanger sequencing technique performed in some recent cases were grouped for analysis. For IDH status, data results from Sanger sequencing by Kansai Molecular Diagnosis Network for CNS tumors for most of the recent cases and IHC in our institute, using IDHR132H antibody, for the other cases ≥ 55 years old were grouped for analysis since the 2016 World Health Organization classification of brain tumors proscribes sequencing for IDH in GBM patients ≥ 55 years old.

### Statistical analysis

For the analysis, χ2, Fisher’s exact and log-rank tests were used for univariate analyses and the Cox proportional hazard model was used for multivariate analysis with p < 0.05 considered as significant. Statistical calculations were performed with IBP SPSS Statistics V25.0 software.

## Results

### Analysis for all 277 newly diagnosed GBM cases

Tables 1 and 2 show various data for all 277 cases, including age; sex; location of the main lesion; side of the lesion (right, left, other side [median, bilateral, multicentric]); KPS; EOR of the lesion; use of intraoperative PDD and fluorescence intensity; use of intraoperative MRI (for removal or for biopsy); IDH and p53 statuses of the lesion; type of RT; type of combination therapy; outcome at discharge (discharge to home, transfer to other facilities or died during hospitalization); and their relationship to patient prognoses. The median OS of the entire 277-case cohort, the 200 non-biopsy cases, and the 77 biopsy cases were 16.6 months, 19.7 months, and 9.7 months, respectively, with GTR (100% of EOR) achieved in 32.9% of the cases. Univariate analysis revealed that younger age, right side, higher KPS, GTR, intraoperative MRI use for removal, p53 status, proton therapy, combination immunotherapy, and discharge to home were good prognostic factors. Cases with IDH mutant status had longer OS (median OS was 28 months) than those with IDH wild-type (median OS was 15 months) although no significant differences were seen.

**Table 1:** Background and diagnostic factors in all 277 cases and their relationship to patient prognoses

Factors		Patient numbers		Median PFS (months)	P values	Median OS (months)	P values
All cases		277		8.7		16.6	
Age	Median (percentile)	66	(57–74)				
	66 or more	149	(53.8%)	8.2	0.334	13.8	0.006
	65 or less	128	(46.2%)	9.4		19.3	
Sex	Men (%)	165	(59.6%)	8.4	0.281	16.6	0.966
	Women	112	(40.4%)	8.7		15.7	
Main Location	Frontal	103	(37.2%)	8.6	0.395	16.2	0.179
	Temporal	93	(33.6%)	8.3		15.1	
	Parieto-Occipital	40	(14.4%)	9.2		24.8	
	Others	41	(14.8%)	9.8		15.7	
Side	Right	132	(47.7%)	9.8	0.030	19.1	0.013
	Left	118	(42.6%)	8.2		15.9	
	Others	27	(9.7%)	5.1		11.0	
KPS	Median (percentile)	70	(60–80)				
	70 or more	190	(68.6%)	9.2	0.190	19.7	0.006
	60 or less	87	(31.4%)	8.6		11.2	
EOR	Non-biopsy	200	(72.2%)	9.5		19.7	
	GTR (100% of EOR)	91	(32.9%)	11.3	0.000	26.5	0.000
	STR	58	(20.9%)	9.2		21.7	
	PR	51	(18.4%)	5.3		11.1	
	Biopsy	77	(27.8%)	7.1		9.7	
5-ALA	Strong	190	(68.6%)	9.1	0.096	16.8	0.300
	Vague	31	(11.2%)	6.7		14.4	
	Positive	11	(4.0%)	11.5		18.0	
	Negative	8	(2.9%)	10.3		13.7	
	Not used/Unknown	37	(13.4%)	4.9		16.7	
Intraoperative MRI	Yes (for removal)	82	(29.6%)	11.2	0.009	21.7	0.000
	Yes (for biopsy)	12	(4.3%)	6.4		8.2	
	Not used	183	(66.1%)	8.1		14.7

**Table 2:** Radiation therapy, combination therapies, and discharge destination outcomes for all 277 cases and their relationship to patient prognoses

Factors		Patient numbers		Median PFS (months)	P values	Median OS (months)	P values
All cases		277		8.7		16.6	
RT	Conventional	195	(70.4%)	9.0	0.000	17.7	0.000
	Hypofractionation	41	(14.8%)	7.3		8.6	
	WBRT	5	(1.8%)	5.5		8.1	
	Proton	33	(11.9%)	11.3		28.7	
	Not used	3	(1.1%)	2.8		2.8	
Combination therapies	TMZ	180	(65%)	8.6	0.001	17.0	0.000
	TMZ + BEV	43	(15.5%)	8.7		11.0	
	TMZ + Immunotherapy	39	(14.1%)	12.5		29.5	
	Others	15	(5.4%)	5.5		10.2	
Place after Discharge	Home	144	(52.0%)	10.5	0.000	23.5	0.000
	Trans	130	(46.9%)	7.1		10.8	
	Death during hospitalization	3	(1.1%)	2.7		2.7	

**Fig. 1:**
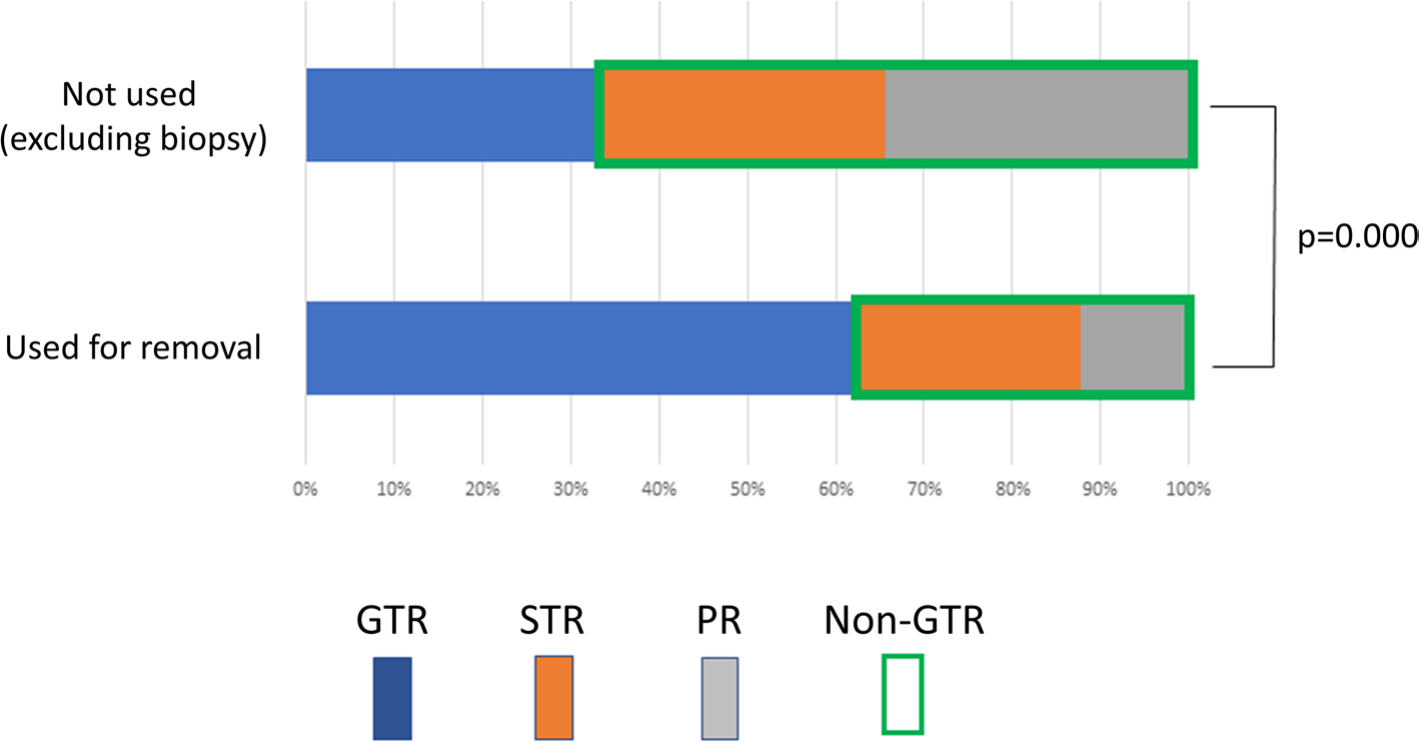
Extent of resection (EOR) of newly diagnosed glioblastoma (GBM) in tumor removal (non-biopsy) cases with and without intraoperative MRI

**Fig. 2:**
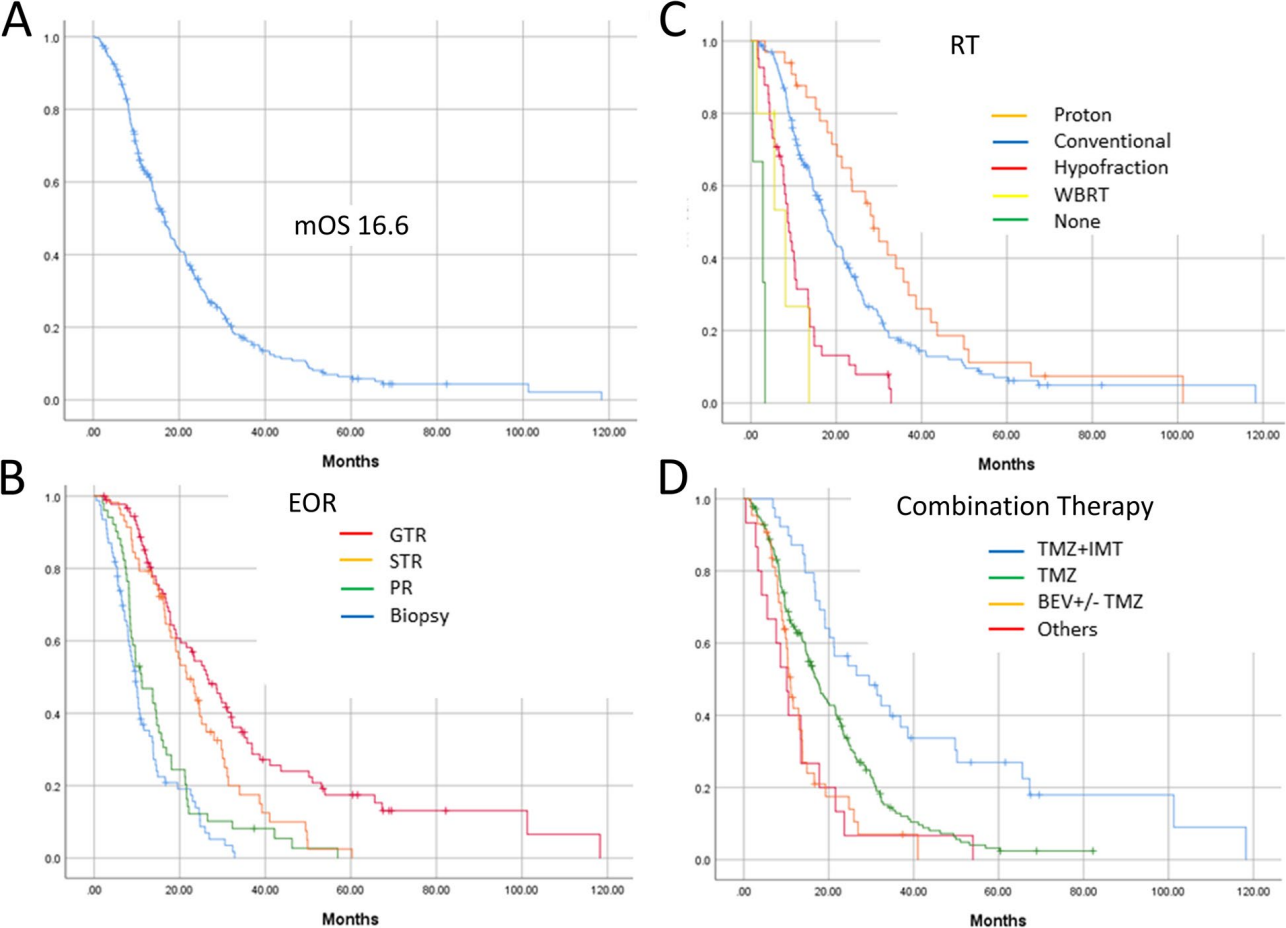
The overall survival (OS) curve for the entire 277 GBM cohort (**A**) and this curve divided by EOR consisting of gross total removal (GTR), subtotal removal (STR), partial removal (PR) and biopsy (**B**); type of radiotherapies (RT), including proton therapy, conventional RT, hypofractionated RT (Hypofraction), whole brain RT (WBRT), and no RT (non) (**C**); and type of combination therapy including temozolomide (TMZ) + immunotherapy (IMT), TMZ, Bevacizumab (BEV) with or without TMZ, and others (**D**)

Table 3 shows the results of a multivariate analysis regarding relationships between OS and 7 factors (65-year-old or less, right side, KPS 70 or more, GTR, p53 negative/wild status, proton therapy, and immunotherapy groups). Intraoperative MRI use was excluded from multivariate analyses as a relating factor of EOR since they were closely related and only 40 (33.6%) out of 119 non-biopsy cases without intraoperative MRI resulted in GTR while 51 (63.0%) out of 81 non-biopsy cases with intraoperative MRI for removal were GTR (p = 0.000, χ2 test) (Fig. 1). In addition, return trips after discharge were also excluded as a confounding factor for patient prognosis and the type of radiotherapy (Conventional/Proton versus Others, p = 0.000, Fisher’s exact test). In the multivariate analysis, GTR, proton therapy, and immunotherapy were the significant prognostic factors (Table 3). Immunotherapy was more often selected for GTR cases than non-GTR cases (p = 0.00, χ2 test) and any interactions between RT and EOR types (p > 0.1, chi-square test) were not found. Figure 2 shows the survival curves comparing each favorable prognostic factor group with the other groups, visually confirming that cases in the GTR and immunotherapy groups show a tailed plateau curve indicative of many long-term survivors. In the multivariate analysis regarding PFS, GTR (p = 0.000, Exp = 1.76) and immunotherapy (p = 0.015, Exp = 1.68) were the significant prognostic factors (detailed data not shown).

**Table 3:** A multivariate analysis of 7 factors from all (277) patient data and their relationship to patient prognoses (OS)

Factors	Groups	P values	Exp (95%CI)
Age	65 or less versus others	0.466	
Side	Right versus others	0.449	
pre-KPS	70 or more versus others	0.093	
EOR	GTR versus others	0.000	2.14 (1.57–2.92)
p53	Negative/wild versus others	0.108	
RT	Proton therapy versus others	0.025	1.60 (1.06–2.40)
Combination therapy	Immunotherapy versus others	0.003	1.89 (1.23–2.88)

### Sub-analysis regarding GTR and non-GTR cases

**Fig. 3:**
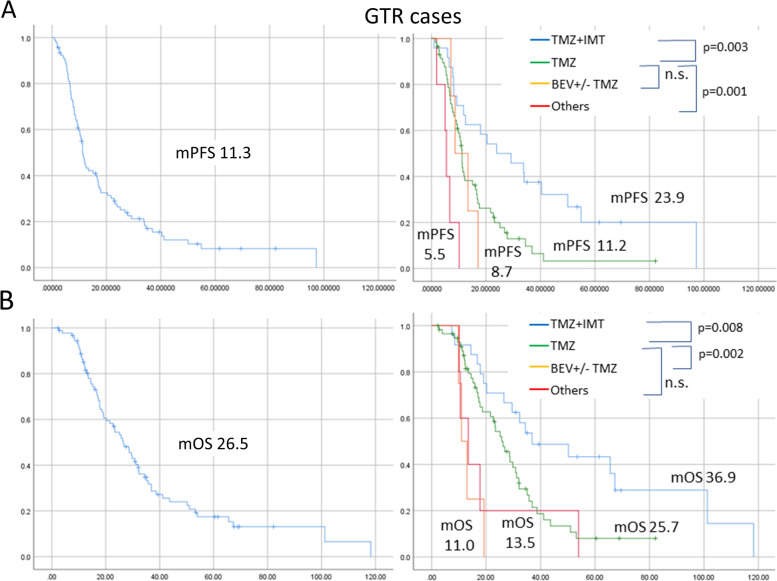
The progression-free survival (PFS) (**A**) and OS (**B**) curves for 91 cases with GTR (left) and each curve of the cases divided by type of combination therapy consisting of TMZ + IMT, TMZ, BEV with or without TMZ, and others (right). The log-rank test was used for analysis

Table 4 shows the results of a multivariate analysis regarding the relationship between OS and candidate factors of 91 GTR cases in order to extract those factors that contributed to the best prognosis in the GTR group. In this analysis, immunotherapy contributed to improvements in prognoses while proton therapy did not contribute despite a lack of interaction between RT and EOR types (p > 0.1, χ2 test). The PFS and OS curves for these factors in the GTR group are shown in Fig. 3. As shown in Fig. 3-B, the median OS was 36.9 months and the 5-year OS% was 43.3% in patients who underwent surgery resulting in GTR and immunotherapy. Table 4 shows the results of a multivariate analysis regarding relationships between OS and candidate factors in 186 non-GTR cases. In this analysis, preoperative KPS and proton therapy, but not immunotherapy, contributed to improvements in prognoses.

**Table 4:** A multivariate analysis of age, KPS, and 2 candidate factors (RT, Adjuvant therapy) in 91 GTR cases and their relationship to patient prognoses (OS), and multivariate analysis in 186 non-GTR cases and their relationships to patient prognoses (OS)

	**Factors**	**Groups**	**P values**	**Exp** **(95%CI)**
GTR cases (91 cases)	Age	65 or less versus others	0.336	
	pre-KPS	70 or more versus others	0.414	
	RT	Proton therapy versus others	0.397	
	Adjuvant	Immunotherapy versus others	0.006	2.35 (1.27–4.33)
Non-GTR cases (186 cases)	Age	65 or less versus others	0.802	
	pre-KPS	70 or more versus others	0.016	1.541 (1.083–2.192)
	RT	Proton therapy versus others	0.023	1.948 (1.094–3.469)
	Adjuvant	Immunotherapy versus others	0.135

### Sub-analysis of immunotherapy cases

To determine any advantages in patient survival, the immunotherapy cases were compared with the control group consisting of patients in better condition (under 76 years old, STR/GTR cases) but with similar patient background, age, tumor side, preoperative KPS, EOR, p53 status, and the use of proton therapy, as shown in Supplementary Table 2. Similarly, the immunotherapy group showed longer survival than the control group (Supplementary Fig. 1).

**Fig. 4:**
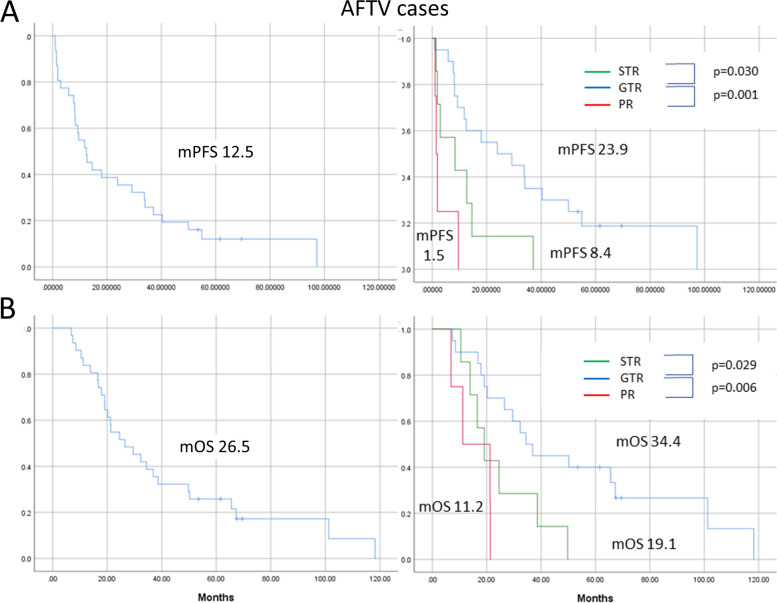
The PFS (**A**) and OS (**B**) curves for 31 cases with AFTV immunotherapy (left) and each curve of the cases divided by EOR consisting of GTR, STR, and PR (right). The log-rank test was used for analysis

Supplementary Table 3 shows the results of a multivariate analysis regarding relationships between OS and candidates for good prognostic factors in the 31 AFTV cases which were the majority (79.5%) of the immunotherapy group. Here, only 3 cases (11%) out of 27 confirmed IDH statuses were IDH mutant type. The median OS of these 3 IDH mutant cases was 29.5 months. As for MGMT status, only 7 cases (28%) out of 25 confirmed statuses were methylated/negative; others were unmethylated/positive. Twenty cases resulted in GTR after surgery, 7 cases were STR, and 4 cases were PR. In this analysis, GTR was a good prognostic factor, in line with results seen in the analysis of the entire immunotherapy cohort. Figure 4 shows the PFS and OS curves for these factors in the AFTV group. Patients with AFTV had a median OS of 26.5 months and the GTR group had a notably good prognosis of 34.4 months for median OS and 40% for 5-year OS%. On the other hand, all 7 STR patients with AFTV died during follow-up periods and their median OS was only 19.1 months.

## Discussion

In this study, younger age, right side, higher KPS, GTR, intraoperative MRI use for removal, p53 status, proton therapy, combination immunotherapy, and discharge to home were good prognostic factors in the univariate analysis. Among these, GTR, proton therapy, and immunotherapy were extracted as good prognostic factors while the use of intraoperative MRI was closely related to EOR. In previous reports, intraoperative MRI was observed to improve the removal rate [18, 19] and our current study is consistent with these reports. As for the right side resulting in a good prognosis in the univariate analysis, the requirement of complex informed consent for advanced therapies, including proton therapy and immunotherapy, may be involved. Informed consent that contained explanations of possible complications tended to include more non-aphasic patients (the majority of whom had right-side lesions) in these advanced therapies. For instance, 20 (61%) out of 33 patients who underwent proton therapy had right-sided lesions as did 24 (61%) out of 39 patients who received immunotherapy (detailed data not-shown).

In the sub-analysis, immunotherapy was a good prognostic factor in the GTR group while GTR was also a good prognostic factor in the AFTV group that happens to represent the majority of immunotherapy given in our institution. Our previous, prospective clinical studies have also shown that high EOR prolongs PFS and OS, although significant differences were only obtained in univariate analyses using the small number of patients that were enrolled in the study [8, 12]. In the present study, however, the significance of this result seems to be high because of the large, albeit retrospective, amount of patient data (277 cases). Moreover, sub-analysis using GTR patient data (Fig. 3) shows immunotherapy can produce long survival (for up to 5 years) in approximately 40% of patients if the tumor lesion is surgically removed without any residual bulk. To the best of our knowledge, no previous study has clarified this phenomenon. On the other hand, the survival curve of the STR group does not indicate the existence of long-term survival cases as shown in Fig. 2 and 4. The significance of immunotherapy in prolonged survival for patients who underwent STR is unclear since there were only 9 cases with immunotherapies (15.5%), including 7 AFTV cases out of 58 STR cases in this study (Supplemental Table 1). However, as an example, all 7 AFTV patients after STR surgery died during follow-up periods and their median OS was less than 20 months, indicating that vaccine therapy, such as AFTV combined with chemoradiotherapy, had no (or minimal) ability to control tumors in patients who had residual tumor bulk after surgery. A recent meta-analysis using 9 total studies, representing 806 GBM patients, showed that half of GBM patients have PD-L1 overexpression, and this expression in tumor tissues is significantly related to a poor OS (HR = 1.63, *P* = 0.003) with heterogeneity (*I*^2^ = 51%) [20]. This result indicates that tumor bulk in most GBM cases engenders resistance to cytotoxic T cell lymphocytes (CTLs), an idea bolstered by our previous studies that showed immunosuppressive PD-1-positive cells and M2 macrophages colocalized to GBM tissue in early relapse cases after AFTV [21, 22]. We therefore speculate that vaccine therapies, including AFTV combined with immune checkpoint inhibitors, M2 macrophage inhibitors, or local therapy that provokes a local immune response, will prolong OS for both GTR and non-GTR cases. Combinations of immunotherapy based on this concept are expected to become the next generation of immunotherapy. [8].

In this study, IDH status was not a statistically significant prognostic factor throughout the entire analysis (Table1) or in the 31 AFTV cases (detailed data not shown). The median OS values of IDH mutant GBM patients were fairly high (28.0 months in the entire analysis and 29.5 months in a sub-analysis using AFTV cases) and we speculate that this is due to the low number of IDH mutant cases. In a recent meta-analysis of GBM, 67 (36.6%) of 183 *IDH1* wild-type GBM cases were PD-L1-positive while only one (3.9%) of 26 *IDH1* mutant GBM cases were PD-L1-positive. [20] The pooled OR indicates that PD-L1 positivity was closely related to *IDH1* status (OR = 9.92, *P* = 0.007) [20], revealing that CTLs in the GBM microenvironment are more effective in IDH1 mutant GBM. Future studies will accumulate the data to confirm this theory. In our study, p53-negative/wild type was a good prognostic factor in the univariate analysis of the entire dataset as shown in Table 1 (median OS values were 19.1, 16.4, and 15.7 months in p53 negative/wild-type, p53 positive/mutant, and unexamined cases, respectively, p = 0.047 by the log-rank test). In this regard, p53 status had no effect on prognosis in the univariate analysis of 238 cases (excluding immunotherapy cases; median OS values were 15.9, 13.7, and 14.7 months in p53 negative/wild-type, p53 positive/mutant, and unexamined cases, respectively, p = 0.162 by the log-rank test) and we assumed that the immunotherapies improved the prognoses of p53 negative/wild-type GBM patients. Our previous studies also suggest that this type is a good prognostic factor in GBM patients who receive AFTV but future prospective studies are needed to verify this.

Limitations of this study must be acknowledged, especially the wide variability of treatment and other factors in this patient population that could affect the results of the multivariate analysis model. Moreover, the sample size for the sub-analysis of AFTV cases shown in Supplementary Table 2 is very small and without an appropriate control group. To address these limitations, a multicenter randomized Phase III trial on AFTV has begun to clarify immunotherapy benefits in GTR patients. Detailed information about MRI data, including the existence of contrast enhancement (CE) in the lesion, volume of CE area, and volume of fluid-attenuated inversion recovery high-intensity areas, were not included in the analysis. Heterogeneity in the type of testing for IDH and p53 and lack of information about MGMT statuses might make the results inaccurate and our insufficient analysis of molecular markers of intratumoral tissues related to prognosis in the immunotherapy group, outside of these 3 markers, should be rectified by additional studies. In addition, as only 3 patients received immunotherapy combined with proton therapy, the effect of this combination cannot be clearly stated in this study.

In conclusion, GTR, proton therapy, and immunotherapy were good prognostic factors in the multivariate analysis of single-center GBM cases. Notably, tumor vaccine therapy for GTR cases achieved high median survival times and long-term survival ratios, revealing that vaccine therapy should be performed for GTR cases.

## Supplementary Information

Additional file 1 Tables and Figures.

## Data Availability

The datasets generated during and/or analyzed during the current study are not publicly available since they include a few unpublished data from original clinical studies but are available from the corresponding author on reasonable request.
