# Supplementary material for: Maximum resection and immunotherapy improve glioblastoma patient survival: a retrospective single-institution prognostic analysis
Source: BMC Neurol. 2021 Jul 19;21:282. doi: 10.1186/s12883-021-02318-1 (PMC8287820; doi:10.1186/s12883-021-02318-1)
Supplement: Supplementary file 1 — Tables and Figures. [file 12883_2021_2318_MOESM1_ESM.doc]

**Maximum resection and immunotherapy improve glioblastoma patient survival: A retrospective single-institution prognostic analysis**

Eiichi Ishikawa^1^, M.D., Ph.D., Narushi Sugii^1^, M.D., Ph.D., Masahide Matsuda^1^, M.D., Ph.D., Hidehiro Kohzuki^1^, M.D., Ph.D., Takao Tsurubuchi^1^, M.D., Ph.D., Hiroyoshi Akutsu^1^, M.D., Ph.D., Shingo Takano^1^, M.D., Ph.D., Masashi Mizumoto^2^, M.D., Ph.D., Hideyuki Sakurai^2^, M.D., Ph.D., and Akira Matsumura^1^, M.D., Ph.D.

**Suppl. Table 1.** Background in biopsy, partial removal, subtotal removal, and gross-total removal cases, and their relationship to various factors.

| Factors |  | Patient numbers |  | Biopsy cases | PR cases | STR cases | GTR cases | P vavue  (Chi-squared test) |
| --- | --- | --- | --- | --- | --- | --- | --- | --- |
| All cases |  | 277 |  | 77 | 51 | 58 | 91 |  |
| Age | 66 or more | 149 | (53.8%) | 58 | 22 | 25 | 44 | 0.000 |
|  | 65 or less | 128 | (46.2%) | 19 | 29 | 33 | 47 |  |
| Sex | Men (%) | 165 | (59.6%) | 50 | 27 | 41 | 47 | 0.066 |
|  | Women | 112 | (40.4%) | 27 | 24 | 17 | 44 |  |
| Main  location | Frontal | 103 | (37.2%) | 31 | 17 | 21 | 34 | 0.000 |
|  | Temporal | 93 | (33.6%) | 12 | 21 | 26 | 34 |  |
|  | Parieto-Occipital | 40 | (14.4%) | 10 | 4 | 8 | 18 |  |
|  | Others | 41 | (14.8%) | 24 | 9 | 3 | 5 |  |
| Side | Right | 132 | (47.7%) | 25 | 28 | 30 | 49 | 0.000 |
|  | Left | 118 | (42.6%) | 36 | 16 | 26 | 40 |  |
|  | Others | 27 | (9.7%) | 16 | 7 | 2 | 2 |  |
| KPS | 70 or more | 190 | (68.6%) | 42 | 32 | 43 | 73 | 0.002 |
|  | 60 or less | 87 | (31.4%) | 35 | 19 | 15 | 18 |  |
| 5-ALA | Strong | 190 | (68.6%) | 42 | 30 | 39 | 79 | 0.002 |
|  | Vague | 31 | (11.2%) | 17 | 6 | 6 | 2 |  |
|  | Positive | 11 | (4.0%) | 3 | 2 | 2 | 4 |  |
|  | Negative | 8 | (2.9%) | 2 | 2 | 2 | 2 |  |
|  | Not used/Unknown | 37 | (13.4%) | 13 | 11 | 9 | 4 |  |
| Intraoperative  MRI | Yes (for removal) | 82 | (29.6%) | 0 | 10 | 20 | 51 | 0.000 |
|  | Yes (for biopsy) | 12 | (4.3%) | 13 | 0 | 0 | 0 |  |
|  | Not used | 183 | (66.1%) | 64 | 41 | 38 | 40 |  |
| IDH | Mutant | 17 | (6.1%) | 1 | 2 | 6 | 8 | 0.201 |
|  | Wild | 197 | (71.1%) | 59 | 34 | 38 | 66 |  |
|  | Not examined | 63 | (22.7%) | 17 | 15 | 14 | 17 |  |
| P53 | Positive/Mutant | 109 | (39.4%) | 27 | 15 | 27 | 40 | 0.154 |
|  | Negative/Wild | 129 | (46.6%) | 34 | 31 | 25 | 39 |  |
|  | Not examined | 39 | (14.1%) | 16 | 5 | 6 | 12 |  |
| RT | Conventional | 195 | (70.4%) | 37 | 43 | 44 | 71 | 0.000 |
|  | Hypofractionation | 41 | (14.8%) | 31 | 4 | 4 | 2 |  |
|  | WBRT | 5 | (1.8%) | 4 | 1 | 0 | 0 |  |
|  | Proton | 33 | (11.9%) | 2 | 3 | 10 | 18 |  |
|  | Not used | 3 | (1.1%) | 3 | 0 | 0 | 0 |  |
| Combination  therapies | TMZ | 180 | (65%) | 40 | 37 | 45 | 58 | 0.000 |
|  | TMZ + BEV | 43 | (15.5%) | 31 | 6 | 2 | 4 |  |
|  | TMZ  + Immunotherapy | 39 | (14.1%) | 0 | 6 | 9 | 24 |  |
|  | Others | 15 | (5.4%) | 6 | 2 | 2 | 5 |  |
| Place  after discharge | Home | 144 | (52.0%) | 24 | 17 | 37 | 66 | 0.000 |
|  | Transfer | 130 | (46.9%) | 51 | 34 | 21 | 24 |  |
|  | Death during hospitalization | 3 | (1.1%) | 2 | 0 | 0 | 1 |  |

**Suppl. Table 2.** A univariate analysis of candidate prognostic factors (age, side, KPS, EOR, p53 and RT) in immunotherapy group and control group consisting of GBM patients under 76 years old who underwent GTR/STR without immunotherapy.

| **Factors** | **Groups** | Immunotherapy* | non- immunotherapy GTR/STR cases (under 76 year-old) | **P value** |
| --- | --- | --- | --- | --- |
|  | All cases | 39 | 102 |  |
| Age | 65 or less | 28 (71.8%) | 56 (54.9%) | 0.085** |
| Side | Right | 24 (61.5%) | 50 (49.0%) | 0.194** |
| pre-KPS | 70 or more | 32 (82.1%) | 79 (77.5%) | 0.849** |
| EOR | GTR | 24 (61.5%) | 60 (58.8%) | 0.849** |
| p53 | Negative/wild | 22 (56.4%) | 40 (39.2%) | 0.088** |
| RT | Proton therapy | 4 (10.3%) | 25 (24.5%) | 0.066** |
| Median OS | Median months (95 percentile) | 29.5 (16.9-42.2) | 23.3 (20.1-26.5) | 0.016*** |

* all patients were under 76 years old, and 6 PR cases are included.

** Fisher’s exact test, *** log-rank test

**Suppl. Figure 1.** Survival curves in the immunotherapy group and control group consisting of GBM patients under 76 years old who underwent GTR/STR without immunotherapy. Blue curve: the immunotherapy group. Red curve: the control group. (p=0.016, log-rank test)
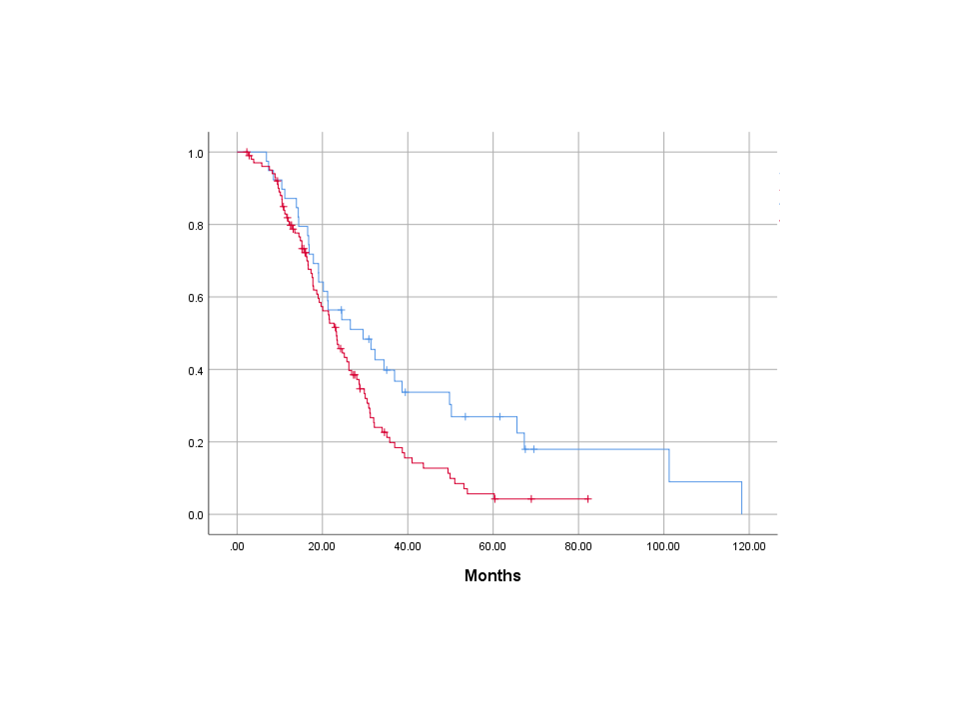


**Suppl. Table 3.** A multivariate analysis of age, KPS and 2 candidate factors in 31 AFTV cases and their relationship to patient prognoses (OS).

| **Factors** | **Groups** | **P values** | **Exp(95% CI)** |
| --- | --- | --- | --- |
| Age | 65 or less versus others | 0.120 |  |
| pre-KPS | 70 or more versus others | 0.471 |  |
| EOR | GTR versus others | 0.005 | 3.64 (1.48-8.93) |
| RT | Proton therapy versus others | 0.780 |  |
